# Artificial Intelligence in Endurance Sports: Metabolic, Recovery, and Nutritional Perspectives

**DOI:** 10.3390/nu17203209

**Published:** 2025-10-13

**Authors:** Gerasimos V. Grivas, Kousar Safari

**Affiliations:** 1 Physical Education and Sports, Division of Humanities and Political Sciences, Hellenic Naval Academy, 18539 Piraeus, Greece; 2 Department of Sport Sciences, Faculty of Education and Psychology, Shiraz University, Shiraz 84334-71946, Iran; parisafari8@gmail.com

**Keywords:** machine learning, sports technology, athlete monitoring, wearable technology, physiological modeling, performance analytics

## Abstract

**Background**: Artificial Intelligence (AI) is increasingly applied in endurance sports to optimize performance, enhance recovery, and personalize nutrition and supplementation. This review synthesizes current knowledge on AI applications in endurance sports, emphasizing implications for metabolic health, nutritional strategies, and recovery optimization, while also addressing ethical considerations and future directions. **Methods**: A narrative review was conducted using targeted searches of PubMed, Scopus, and Web of Science with cross-referencing. Extracted items included sport/context, data sources, AI methods including machine learning (ML), validation type (internal vs. external/field), performance metrics, comparators, and key limitations to support a structured synthesis; no formal risk-of-bias assessment or meta-analysis was undertaken due to heterogeneity. **Results**: AI systems effectively integrate multimodal physiological, environmental, and behavioral data to enhance metabolic health monitoring, predict recovery states, and personalize nutrition. Continuous glucose monitoring combined with AI algorithms allows precise carbohydrate management during prolonged events, improving performance outcomes. AI-driven supplementation strategies, informed by genetic polymorphisms and individual metabolic responses, have demonstrated enhanced ergogenic effectiveness. However, significant challenges persist, including measurement validity and reliability of sensor-derived signals and overall dataset quality (e.g., noise, missingness, labeling error), model performance and generalizability, algorithmic transparency, and equitable access. Furthermore, limited generalizability due to homogenous training datasets restricts widespread applicability across diverse athletic populations. **Conclusions**: The integration of AI in endurance sports offers substantial promise for improving performance, recovery, and nutritional strategies through personalized approaches. Realizing this potential requires addressing existing limitations in model performance and generalizability, ethical transparency, and equitable accessibility. Future research should prioritize diverse, representative, multi-site data collection across sex/gender, age, and race/ethnicity. Coverage should include performance level (elite to recreational), sport discipline, environmental conditions (e.g., heat, altitude), and device platforms (multi-vendor/multi-sensor). Equally important are rigorous external and field validation, transparent and explainable deployment with appropriate governance, and equitable access to ensure scientifically robust, ethically sound, and practically relevant AI solutions.

## 1. Introduction

Endurance sports impose sustained physiological challenges, particularly in energy metabolism, hydration, recovery, and nutritional regulation [1]. Optimizing these domains has long been central in applied sports science and clinical nutrition. In recent years, the proliferation of real-time data from wearables, metabolic analyzers, and training apps has increased the demand for tools capable of interpreting and applying such data in individualized ways [2].

Artificial intelligence (AI), including machine learning (ML), deep learning, and other data-driven algorithms, has emerged as a transformative force in performance science and healthcare [3]. In endurance sports, AI supports not only monitoring but also predictive and personalized interventions across domains such as training load, metabolic stress, macronutrient periodization, hydration, and recovery [4]. This is particularly relevant in sports like marathon, triathlon, and cycling, where minor physiological fluctuations can disproportionately affect performance and health outcomes [5,6].

By integrating multi-source data including heart rate variability (HRV), sleep, nutrition, and glucose responses, AI enables precision insights that surpass traditional coaching or static dietary plans [7]. Early studies have demonstrated promising applications in fatigue prediction, real-time fueling strategies, and nutrition planning based on metabolic profiling [8].

Prior reviews have addressed specific subdomains using AI for precision nutrition [9], continuous glucose monitoring in sport [10], inertial measurement unit (IMU)-based running-gait assessment [11], and wrist-worn heart-rate validation methods [12], but an endurance-specific synthesis that links data modalities and AI methods to practice and governance across metabolic health, recovery monitoring, and nutrition/supplementation personalization remains limited. Accordingly, this narrative review synthesizes current AI applications in endurance settings across those pillars, maps reported performance and validation status (distinguishing internal vs. external/field and typical comparators), distinguishes what is actionable now from what requires further validation, and outlines practice-facing workflows and governance considerations [13,14,15].

## 2. Background

### 2.1. What Is Artificial Intelligence (AI)

Operationally, AI is the study and engineering of computational agents that perceive their environment and act to achieve specified goals under uncertainty (often formalized as the study of intelligent agents) [16]. While early visions emphasized human-like cognition (learning, reasoning, problem solving), most contemporary systems—especially ML and deep learning—optimize task performance via statistical learning, optimization, and searching rather than explicitly simulating how humans think. In sport and exercise science, AI is increasingly applied to process large, complex datasets that exceed the capabilities of conventional statistical methods [16].

Core AI techniques include ML, data-driven methods that learn predictive patterns from examples, and deep learning, which uses multi-layer neural networks to learn hierarchical representations [16,17]. In practice, ML enables data-driven decision-making from patterns identified in historical and real-time information [18], whereas deep learning models complex relationships, particularly within high-dimensional data [19]. Most sport-related applications fall under narrow AI, focusing on specific tasks such as predicting fatigue or classifying movement patterns [20]. A key strength is multimodal integration combining physiological, biomechanical, behavioral, and contextual data into unified analytical frameworks, which enables insight into how variables interact over time and under varying training conditions [16,21]. This multidimensional data fusion offers novel opportunities for applied decision support in endurance sport [22].

Explainable artificial intelligence (XAI) refers to methods that make the decisions and internal logic of AI systems understandable to humans [23]. These approaches, including interpretable-by-design models and post hoc explanation methods, aim to improve transparency and interpretability for end users, which is particularly relevant in applied sport settings where trust, clarity, and usability are critical [23,24]. The use of AI represents a paradigm shift in how endurance performance is analyzed and understood, moving from isolated measurements to integrated, adaptive systems capable of capturing the dynamic nature of human performance.

### 2.2. How AI Is Used in Endurance Sports

**Figure 1:**
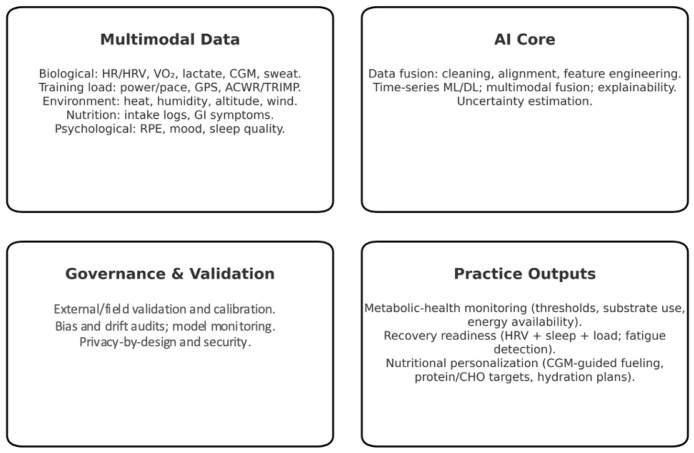
Conceptual overview of AI in endurance sports.

The application of AI is increasingly observed in endurance sports to support decision-making, automate data interpretation, and enhance the precision of training strategies. Athletes in disciplines such as marathon running, cycling, triathlon, and ultra-endurance events generate large volumes of performance-related data. AI tools enable coaches and athletes to process these complex datasets efficiently and translate them into practical, actionable insights (Figure 1) [3,25].

A major area of application involves optimizing training and recovery. Algorithms process data from wearables including heart rate monitors, GPS devices, and accelerometers to identify trends in performance, detect signs of fatigue, and estimate recovery needs. In a 12-week longitudinal study of endurance athletes (*n* = 43; 3572 athlete-days), ML models using HRV together with training, sleep, diet and wellness measures predicted next-morning perceived recovery status and day-to-day HRV change, outperforming an intercept-only baseline [6].

In addition, the use of AI supports the refinement of nutrition and fueling strategies. During prolonged endurance exercise, ML models trained on noninvasive physiological signals and sweat biomarkers (heart rate, core temperature, sweat sodium concentration, whole-body sweat rate) have predicted hydration status as percent body-mass loss, informing individualized fluid-replacement strategies [26]. Model performance was reported with standard agreement statistics (e.g., RMSE and Bland–Altman limits of agreement), enabling field interpretation of percent body-mass loss predictions [26]. In parallel, ML models that combine power output and heart rate (HR) with session characteristics and environmental conditions estimate in-session carbohydrate utilization and total energy expenditure in running and cycling, offering practical targets for carbohydrate availability and timing [6]. Although continuous glucose monitoring (CGM)-based predictive models for individualized postprandial glycemic responses were developed in general-population cohorts, they demonstrate clear translational relevance for athlete-specific fueling decisions [27,28]. Meanwhile, CGM is increasingly used in endurance sport, but consensus on interpretation and actionable protocols remains limited [10]. Because CGM signals exhibit physiological lag and device-dependent error, AI-derived fueling decisions (e.g., gel spacing, g·h^−1^ carbohydrate targets) should include uncertainty bounds and be read in light of validation metrics and session context (intensity, temperature, prior intake).

In performance planning, simulation-based optimization (optimal control/dynamic programming) has been used to derive course-specific pacing policies that account for gradient, wind, and physiological constraints; in practice, these course-aware plans outperform fixed-power pacing in projected time-trial performance [29,30]. In recreational marathon runners, El Dandachi et al. [31] analyzed multiphysiological time-series from nine athletes using a variational autoencoder (VAE) with Lyapunov-based stability analysis to anticipate the “marathon wall”, providing a proof-of-concept for individualized pacing guidance from cardio-GPS data. Beyond pacing, ML methods in biomechanical analysis detect gait asymmetries, fatigue-related changes in running mechanics, and injury-risk indicators using IMUs with force-plate–derived ground truth [32,33,34]. Subject-specific classifiers trained on wearable signals outperform group-based models for identifying fatigue-related alterations in running (≈68–69% vs. 57–62%) [33]. Deep-learning models also estimate multidimensional GRF/M from wearable accelerometry with high agreement to force-plate ground truth [34]. In addition, a systematic review supports the validity and reliability of IMU-based running-gait assessment in adults [32]. Because several outputs in this section rely on indirect sensing (e.g., wrist-PPG vs. ECG; IMU-to-GRF inference; CGM with physiological lag), accuracy is task- and modality-dependent; interpretation should follow the agreement statistics reported in each study [35].

In psychological monitoring, Duan et al. [36] proposed a hybrid transformer–boosting classifier (BERT-XGBoost) that identifies athletes’ psychological states (e.g., anxiety, stress, emotional balance) from annotated self-reports and observational logs, reporting 94% overall accuracy under internal validation. Complementarily, Zhang et al. [37] modeled athlete engagement from questionnaire-derived features (cohesion, passion, mental toughness) in *n* = 445 athletes (ages 17–22; multiple team sports) with 5-fold cross-validation; a support vector regression (SVR) model optimized via particle swarm optimization (PSO) achieved accuracy of 0.9262 (RMSE, 0.1227; mean squared error [MSE], 0.0146; mean absolute error [MAE], 0.0656), reducing errors relative to the strongest baseline model and modestly increasing the coefficient of determination (R^2^).

Across these exemplars, AI models exceeded intercept-only or conventional baselines on standard metrics (e.g., RMSE/MAE, accuracy, calibration), indicating practical uplift in prediction and decision support under field conditions [6,33,34]. While applications in endurance sport are still maturing, multiple lines of evidence indicate a shift toward multidimensional, data-driven coaching that integrates physiological, biomechanical, environmental, and behavioral data (Table 1). Examples include recovery prediction from HRV combined with training/sleep/diet logs [6], course-specific pacing via simulation-based optimization [29,30], wearable-based biomechanical inference (fatigue classification and GRF/M estimation) [33,34], and CGM-informed personalized fueling models [27,28], with psychological/engagement modeling providing additional context [37].

Compared with traditional heuristics and single-sensor thresholds, AI-driven techniques show quantitative advantages under field conditions. In recovery prediction, ML models trained on HRV together with training, sleep, diet, and wellness measures outperformed an intercept-only baseline for both perceived recovery and day-to-day HRV change [6]. In fueling/hydration, models using noninvasive biosignals and sweat biomarkers predicted percent body-mass loss with reported RMSE and Bland–Altman agreement, enabling actionable, uncertainty-aware prescriptions [26]. For race planning, simulation-based optimal control/dynamic programming generated course-specific pacing plans that outperformed fixed-power or equal-speed heuristics in projected time-trial performance [29,30]. In running biomechanics, subject-specific wearable-signal classifiers surpassed group models for fatigue-related gait changes (≈68–69% vs. 57–62%), and deep learning on accelerometry estimated multidimensional GRF/M with high agreement to force-plate ground truth, exceeding simpler regression baselines [33,34]. Together with CGM-informed nutrition models [27,28] and psychological-state modeling [36,37], these results illustrate practical uplift over conventional methods and motivate data-driven coaching workflows.

### 2.3. Data Sources and Modalities for AI in Endurance Sports

The effectiveness of AI in endurance sports relies heavily on the integration of diverse and complementary data sources. These datasets reflect the multifactorial nature of endurance performance, spanning physiological, environmental, nutritional, psychological, and training-related dimensions [6,27,28,29,30,32,33,34,37].

Biological data serve as the physiological foundation of AI systems. Common parameters include HR, oxygen uptake (VO_2_), lactate threshold, glucose levels, and HRV. These metrics are typically collected through laboratory testing or wearable devices and are critical for assessing internal load, cardiorespiratory stress, and metabolic state [38,39].

Environmental data provide important context for interpreting performance outputs [40,41]. Models based on AI that explicitly incorporate temperature, humidity, wind speed, and solar radiation have quantified their impact on endurance performance; in a dataset of 1258 races (7867 athletes), decision-tree models achieved R^2^ = 0.21–0.58 and identified an optimal ~10–17.5 °C air-temperature range, with performance declining by 0.3–0.4% per °C outside this band [42]. In parallel, data-driven optimization models that include gradient, wind, and course profile improve course-specific pacing projections [29,30].

Nutritional data, including food intake logs, supplement use, and glucose variability measured via continuous glucose monitoring, enable AI to account for energy availability and fueling status. When aligned with training and metabolic demands, such data support more precise dietary strategies, with AI models predicting individualized postprandial glycemic responses and informing tailored recommendations [43,44].

Training-related data encompass session duration, frequency, intensity, accumulated workload (e.g., TRIMP, ACWR), and recovery status, commonly derived from GPS devices, accelerometers, and training platforms. These metrics enable computation of acute and chronic load indices and serve as inputs to AI models that forecast next-day recovery in endurance athletes and predict running performance from training logs [6,45].

Psychological and subjective data such as ratings of RPE, mood states, sleep quality, and mental fatigue are increasingly incorporated into ML pipelines in endurance sport. In distance runners, smartwatch-derived signals (e.g., HR, cadence, stride/velocity metrics) have been used to classify RPE levels with ~85% accuracy across trained and untrained participants and to estimate instantaneous RPE during outdoor 5 km efforts (root mean square error ≈ 1.8 with subject-independent models; ≤0.45 with brief individual calibration) [46,47]. Incorporating these variables provides information beyond load-only features and supports individualized monitoring.

The power of AI lies in its ability to synthesize heterogeneous inputs into athlete-specific models. In trained cyclists, Barsumyan et al. [48] used ML on cardiovascular drift and aerobic decoupling from standardized 60 min rides at ~75% functional threshold power to classify training response (responder vs. non-responder) across repeated monthly tests; cross-validated accuracy ranged 0.87–0.93 (best: variational Gaussian process). Wang et al. [49] trained supervised ML models on inertial measurement unit data to discriminate fatigued from non fatigued states. In complementary experiments with adult runners on a treadmill (*n* = 16) at or around maximal lactate steady state, as well as adult runners in prolonged outdoor runs (*n* = 9), subject-specific inertial measurement unit-based classifiers outperformed group models with accuracy of about 68 to 69% versus 57 to 62% [33]. Deep learning approaches further support this direction. Chang et al. [50] studied healthy male runners (*n* =19), and applied convolutional neural network, long short term memory, attention, and a convolutional neural network plus long short term memory hybrid to multi inertial measurement unit raw time series and achieved effective classification of pre, mid, and post fatigue states, with the hybrid architecture outperforming single model baselines.

In practice, biological and nutritional domains are interdependent rather than siloed. AI systems fuse biological inputs (e.g., HRV, VO_2_ thresholds, lactate/CPET metrics, recovery status), nutritional inputs (diet logs, CGM traces, sweat rate and sodium concentration), and context (training load, sleep, environment) to produce session- and athlete-specific prescriptions; e.g., carbohydrate targets (g·h^−1^ and timing), fluid/sodium plans, and recovery-protein goals [9,26,27,28,42,51]. For example, models that combine power/pace and weather with CGM and recent intake can forecast glycemic excursions to guide gel spacing and drink composition [27,28,52], while CPET-derived thresholds integrated with workload and heat index refine carbohydrate-oxidation estimates and hydration strategies [5,42,51,53]. Modality-specific accuracy and cross-device comparability constrain downstream modeling; outputs should be interpreted alongside the reported error/agreement statistics in each dataset (e.g., RMSE/MAE/κ, Bland–Altman limits of agreement) [12,54,55].

## 3. Methods

A narrative review was undertaken with targeted searches in PubMed/MEDLINE, Scopus, and Web of Science plus citation tracking. Eligibility included peer-reviewed human studies or reviews in endurance contexts that used AI/ML or directly informed AI-enabled workflows; non-English, animal/pediatric studies, device engineering without applied outcomes, and non-methodological commentaries were excluded. Screening was conducted in two stages. Extracted items covered sport/context, data sources/sensors, AI methods, including ML, validation type (internal vs. external/field), metrics, comparators, and limitations. Given heterogeneity, evidence was organized as a structured narrative synthesis by application domain and data modality; validation type and headline metrics were reported where available. No formal risk-of-bias assessment or meta-analysis was performed. Where available, error/accuracy metrics—including root-mean-square error (RMSE; square root of the mean squared prediction error, in the outcome’s units; lower is better and more sensitive to large errors than MAE), MAE, accuracy, κ, and Bland–Altman limits of agreement—were extracted, and the interpretive limits of indirect/derived measurements were noted.

## 4. Implications for Metabolic Health, Recovery, and Nutrition

### 4.1. AI and Metabolic Health

Metabolic health, defined as normal insulin sensitivity and glycemic control, a favorable lipid profile, normal blood pressure, and a healthy adiposity distribution [56], is foundational to endurance performance, affecting energy availability, substrate utilization, mitochondrial efficiency, and recovery capacity. In recent years, AI has enabled more dynamic assessments of these domains by integrating physiological signals from wearables, continuous glucose monitors (CGMs), and cardiopulmonary exercise testing (CPET) into predictive models [6,27,43]. Practical outputs include session-specific carbohydrate targets (g·h^−1^ and timing), fluid and sodium plans tailored to heat/humidity and individual sweat profile, and recovery-protein goals, with updates as new data accrue [9]. Reported performance for non-invasive metabolic profiling typically includes single-digit mean absolute error (MAE) and high correlations to laboratory standards (e.g., r ≈ 0.97–0.99 for threshold detection) [57,58], indicating good agreement under controlled conditions while underscoring the need for external, endurance-specific validation.

A key advancement is the application of ML to glucose regulation and fuel usage. Systems like Supersapiens, originally designed for clinical diabetes management, are increasingly adopted by endurance athletes to visualize glucose trends in real time. This facilitates more precise carbohydrate timing during training and racing, tailored to individual glycemic responses [52,59].

The use of AI has also been extended to the estimation of metabolic thresholds, including ventilatory threshold (VT) and lactate threshold (LT), based on non-invasive data. Zignoli et al. [57] demonstrated that recurrent neural networks can estimate VT1 and VT2 with mean absolute errors below 10%. Similarly, the Oxynet platform applied deep learning to over 1200 CPET datasets, including data from both healthy individuals and patients with cardiopulmonary diseases, achieving near-expert agreement in threshold detection (r ≈ 0.97–0.99) [58]. These models reduce reliance on laboratory-based testing and offer scalable solutions for metabolic profiling.

Recent studies have expanded this potential further. For example, Sheridan et al. [60] developed ML models using HR and IMUs to estimate VO_2_ during intermittent team sports, achieving RMSE values around 5 mL·min^−1^. Other approaches have used photoplethysmography (PPG) from wrist-worn sensors to estimate VO_2_ continuously via random forest algorithms [61], showing promise for non-laboratory monitoring. Because these are indirect estimates (inferred from HR/IMU/PPG rather than breath-by-breath gas exchange), interpretation should consider the reported RMSE/MAE and correlations to laboratory gold standards, as well as modality-specific sources of error [35]

Collectively, these applications allow athletes and coaches to monitor energy system dynamics, substrate shifts, and fatigue-related metabolic changes with unprecedented granularity. These metabolic insights inform day-to-day decisions on recovery and nutrition, keeping prescriptions aligned with each athlete’s evolving physiology.

### 4.2. AI in Recovery Monitoring and Readiness Assessment

Recovery is a critical determinant of endurance performance, affecting not only adaptation but also long-term athletic sustainability [62]. One of the most established recovery metrics is HRV, a marker of autonomic nervous system function. AI systems incorporate HRV alongside contextual inputs such as training load, sleep quality, and psychological status to create individualized readiness scores. For example, AI-driven random-forest models trained on IMU-derived biomechanical features distinguished no/mild/heavy fatigue during outdoor track running (leave-one-subject-out validation), with accuracy ranging from 0.761 (single left tibia) to 0.905 (all sensors) [63].

Beyond HRV, AI applications now synthesize additional biometric signals such as resting HR, respiratory rate, sleep architecture, and skin temperature captured via wearable devices. These multimodal datasets allow for more nuanced assessments of recovery trajectories. For example, Miller et al. [64] reported epoch-by-epoch agreement of 89% for two-state (sleep/wake) classification (sleep sensitivity 95%, wake specificity 51%, κ = 0.49) and 64% for four-stage sleep (κ = 0.47) when benchmarking WHOOP against polysomnography.

In recovery classification, ML models have further advanced the field. In this domain, ML models trained on IMU signals can discriminate fatigue states during outdoor running; for example, random-forest classifiers achieved accuracies from 0.76 (single tibial sensor) to 0.90 (multi-sensor) under leave-one-subject-out validation [63]. Similarly, deep learning models using raw multi-IMU time-series (CNN/LSTM/attention) classify pre-/mid-/post-fatigue stages, with a hybrid CNN + LSTM outperforming single-model baselines [50]. Despite these innovations, several limitations remain. Sensor-derived metrics can be affected by external noise, including movement artifacts, device placement, or skin characteristics [65].

Nevertheless, the adaptive nature of AI-based recovery models, capable of updating daily based on an athlete’s evolving physiological state, marks a shift from traditional, one-size-fits-all approaches. These tools can support more informed decisions regarding session intensity, rest periods, and tapering strategies, ultimately promoting athlete resilience and minimizing injury risk [6,62,66].

### 4.3. AI in Nutritional Supplementation and Metabolic Optimization

In endurance athletes, AI is increasingly used to optimize nutritional supplementation and enhance metabolic adaptations [67]. The ability of AI algorithms to analyze complex interactions among supplements, individual metabolic responses, training loads, and recovery states facilitates highly personalized strategies aimed at improving endurance performance and effective recovery [9].

Emerging research demonstrates that AI-driven systems can recommend precise supplementation dosages and timing tailored to individual metabolic profiles. Evidence-based supplementation can modulate post-exercise muscle-damage and inflammatory responses in endurance athletes. In particular, appropriate provision of protein and selected amino-acid strategies (e.g., creatine, branched-chain amino acids) has been associated with attenuated rises in creatine kinase and improved recovery markers, supporting individualized recovery protocols [68,69]. Within endurance sport, AI is increasingly used to personalize nutritional supplementation and metabolic adaptations by integrating genotype, wearable/CGM signals and contextual training data [9]. Because many nutrition signals are indirectly measured (e.g., CGM with physiological lag, wrist-PPG, self-reported intake), interpretation should account for device- and context-specific error; exemplar evaluations in swimming show substantial degradation in wrist-PPG HR during water-based activity (+13–56 bpm mean deviation; +49–52 bpm SD) [10,27,28].

Moreover, recent studies, including those by Burke et al. [70], have highlighted the complex relationship between dietary interventions and metabolic responses in elite endurance athletes. Burke’s study revealed that despite substantial increases in fat oxidation with a ketogenic low-carbohydrate, high-fat diet (LCHF), athletes experienced impaired performance due to reduced exercise economy at high intensities [70]. Furthermore, integrating real-time wearable data (e.g., continuous glucose monitoring) with AI enables dynamic nutritional adjustments during endurance events, promoting sustained performance through optimized glucose management [71]. AI-driven nutritional guidance could prevent such mismatches by identifying athletes who might negatively respond to specific macronutrient strategies, thus personalizing supplementation and dietary recommendations accordingly.

Data-driven pipelines are being developed to synthesize large experimental and real-world datasets on ergogenic supplementation, with the goal of characterizing response heterogeneity and informing individualized decisions [9]. Despite these advancements, significant challenges persist. Data accuracy, consistency, and ethical considerations regarding data ownership and commercial biases in supplement recommendations require attention. Future research should prioritize robust validation studies, transparent and explainable algorithms, and the collection of inclusive datasets that represent diverse athletic populations.

In summary, AI-powered nutritional supplementation offers substantial promise for metabolic optimization and performance enhancement in endurance athletes. Nevertheless, continued interdisciplinary collaboration and rigorous scientific validation remain crucial for translating these technological innovations into practical, ethical, and widely accessible tools.

### 4.4. AI-Powered Nutritional Personalization

AI is reshaping nutrition science in endurance sports, offering unprecedented opportunities for real-time, individualized fueling strategies. Traditional dietary recommendations often fail to account for personal variability in metabolism, exercise intensity, and gastrointestinal tolerance. In contrast, AI systems leverage physiological, behavioral, and contextual inputs to optimize energy intake and dietary periodization.

A key innovation involves the integration of continuous glucose monitoring (CGM) with ML models to guide carbohydrate timing. For instance, Ishihara et al. [72] demonstrated that glucose stability during a 160 km ultramarathon was associated with superior pacing and faster finishing times. Such findings highlight the value of real-time feedback in personalizing in-race fueling especially when supported by adaptive algorithms that learn from an athlete’s historical and contextual data.

Beyond glucose, AI tools enhance dietary tracking accuracy. Convolutional neural networks (CNNs) applied to food image recognition have achieved over 90% accuracy in identifying food types and estimating nutrient content from smartphone photos [73]. This reduces user burden and improves adherence two common challenges in long-term nutrition monitoring.

Mobile apps now incorporate AI to deliver context-aware guidance, adapting meal recommendations based on physical activity levels, glycemic responses, and user preferences. The nBuddy app, for example, combines CGM inputs with ML to personalize meal timing and content throughout the day. A recent intervention showed improved glycemic control and dietary compliance among users over a 3-month period [74].

Gastrointestinal (GI) discomfort is common in endurance events and relates to intake patterns and prior GI history [75]; management emphasizes modifiable factors (e.g., composition/osmolality of intake, hydration practices, heat) and individualized “gut-training” approaches [76]. Within endurance sport, AI is increasingly used to integrate genotype, wearable/CGM signals, and training-load context for personalized nutrition strategies [9].

Despite these advances, several limitations remain. Many AI platforms depend on high-fidelity input data, and errors in CGM readings, inconsistent food logging, or inter-individual variability in digestion and metabolism can reduce algorithm reliability. Moreover, privacy concerns are growing, especially as nutrition-focused AI tools increasingly operate within commercial ecosystems that monetize personal health data [77].

In conclusion, AI-powered nutritional personalization has the potential to transform endurance nutrition moving from generic advice to precision-based, real-time interventions. Future developments must emphasize algorithm transparency, robust validation, and athlete education to ensure that these tools are both effective and ethically sound (Table 2).

## 5. Ethical Considerations in Endurance Sports AI

### 5.1. Data Privacy and Informed Consent

As AI becomes embedded in endurance sport, wearables and digital platforms collect sensitive biometric and contextual data (e.g., HRV, glucose, sleep architecture, geolocation). Athletes often lack clear, accessible information about how these data are stored, shared, retained, or repurposed, increasing privacy risk.

A central concern is opacity around data ownership and secondary use: consumer technologies frequently rely on vague terms of service that permit reuse of (even “anonymized”) data for commercial or research purposes without meaningful user engagement [78]. Regulatory frameworks such as the GDPR require informed consent, purpose limitation, and user control, but implementation is inconsistent and privacy notices are often dense or technical, limiting comprehension [79]. In team or institutional settings, additional power asymmetries arise when coaches, staff, or sponsors access athlete data; youth, amateur, or contract-dependent athletes may feel compelled to agree without fully understanding the implications [80].

Practical safeguards include data minimization with clear purpose specification; default-off data sharing; explicit retention schedules and audit trails; role-based access controls; athlete-facing dashboards that show what is collected, by whom, and why; portable data exports and deletion options; and dynamic (ongoing) consent so permissions can be revised as contexts change. Consent should be treated as a continuing process—not a one-time checkbox. Implement data minimization with default-off sharing, role-based access controls, explicit retention schedules, and dynamic consent surfaced in athlete-facing dashboards. Conduct a Data Protection Impact Assessment before onboarding any new AI platform.

### 5.2. Algorithmic Transparency and Bias

A central ethical challenge is limited algorithmic transparency. Many ML/deep learning models operate as “black boxes”, producing recommendations without a clear account of their internal logic—this is problematic when outputs influence training decisions, recovery protocols, or risk assessments where trust, accountability, and human oversight are essential [81]. Lack of interpretability can erode confidence; inconsistent or conflicting feedback across platforms further confuses athletes and coaches when the provenance of a recommendation cannot be traced [82,83]. In elite settings, this can translate into misguided programming or misdiagnosed fatigue with real consequences for performance and athlete welfare [84].

Closely related is algorithmic bias from non-representative training data. Models over-exposed to elite, Western, or male cohorts may underperform for female, recreational, older, or ethnically diverse athletes. Even basic biosignals (e.g., HR, SpO_2_) can vary in accuracy with skin tone, compounding disparities in downstream insights [65,85]. Bias can also be introduced during data selection, labeling, and preprocessing, especially when most data come from high-performance environments thereby reproducing existing inequalities in access to sports science and technology [86,87].

Practical safeguards include pre-specifying model purpose and target populations; conducting external/field validation and calibration with stratified performance reporting (by sex, age, skin tone, performance level); performing routine bias and drift audits; quantifying and communicating predictive uncertainty in athlete-facing outputs; applying XAI—using interpretable-by-design models where feasible and post hoc explanation tools otherwise—to reveal the main features driving recommendations [82]; publishing transparent “model cards” and “data sheets” that disclose training-data characteristics and limits; and maintaining multidisciplinary, athlete-inclusive development with human-in-the-loop oversight for consequential decisions. Prioritizing transparency-by-design and representative datasets is essential if AI is to become a trusted and equitable partner in endurance-sport decision-making. Report stratified performance and calibration (sex, age, skin tone, performance level), quantify uncertainty in athlete-facing outputs, and use interpretable-by-design models where feasible with XAI otherwise. Publish model cards and data sheets detailing training data, limitations, and update cadence.

### 5.3. Commercialization and Conflicts of Interest

The expanding role of AI in endurance sport is tightly coupled to commercial incentives. Technology vendors, wearable manufacturers, and digital-health platforms drive innovation to enhance performance—and to grow market share, engagement, and data assets. Popular systems (e.g., WHOOP, Boston, MA, USA; Garmin Connect, Supersapiens) are marketed as performance tools, yet their business models often depend on continuous collection of biometric and behavioral data to power proprietary algorithms [88,89]. While these platforms can deliver useful feedback and individualized recommendations, product design is frequently steered by subscription economics, stickiness metrics, and long-term data retention. This raises concerns about data ownership, secondary use, and transparency, with terms of service that many athletes cannot realistically audit or understand [90].

Commercial pressures can also create conflicts of interest. Features that prioritize engagement or upselling, such as prominent stress or recovery scores, may outpace physiological validation, potentially misleading users or prompting unnecessary behavior changes. This dynamic of “data capitalism”, whereby athlete-generated data are monetized, conflicts with the beneficence expected in sport science and health technology [91].

Reliance on opaque, proprietary algorithms can shift the athlete–coach relationship toward passive compliance, and cost barriers for premium devices or subscriptions risk widening access gaps across programs and regions [92].

Practical safeguards are therefore essential. Athlete-centered data rights (clear ownership, portability, meaningful opt-in/opt-out), plain-language privacy policies, and data-minimization with privacy-by-design should be the default. Independent validation of algorithms, public model and dataset summaries (“model cards”/“data sheets”), disclosure of commercial interests, and firewalls between engagement metrics and health-critical outputs help maintain scientific integrity. Open or auditable interfaces and transparent update logs support accountability, while tiered pricing, institutional licensing, and community access programs can reduce inequities. Progress will require coordination between vendors, teams, regulators, and researchers so that commercialization does not override athlete welfare, scientific validity, or ethical accountability. Require independent external validation of vendor algorithms and firewall engagement metrics from health-critical outputs. Provide plain-language data rights (ownership, portability, opt-in/opt-out) and offer tiered pricing or institutional licenses to reduce access inequities.

### 5.4. Accessibility and the Digital Divide

While AI holds transformative potential for endurance sports, its benefits are not equitably distributed. Many AI-powered tools such as advanced wearables, personalized nutrition platforms, and subscription-based analytics services remain inaccessible to a large portion of the athletic population, especially in low-resource settings or among recreational athletes. This growing disparity is increasingly described as the digital divide in sports performance science [93].

High-end wearables (e.g., WHOOP, Boston, MA, USA; Supersapiens; Stryd) and premium software platforms (e.g., TrainingPeaks, Louisville, CO, USA; Athletica.ai) often require substantial financial investment and digital literacy. Athletes from underrepresented regions, older age groups, or disadvantaged socioeconomic backgrounds may lack the infrastructure (e.g., reliable internet, compatible devices) or the technical skills necessary to engage with these technologies effectively. As a result, performance-enhancing interventions may disproportionately benefit more privileged groups, reinforcing existing inequalities in sport participation and support systems [80].

In addition, the design of many AI systems reflects the needs and data of elite, Western athletes, leading to models that may not generalize well across diverse populations. Algorithms trained on homogeneous datasets can overlook variations in body composition, training environments, and physiological norms. This bias reduces the validity and applicability of AI-driven recommendations, potentially excluding underserved groups from accurate insights and meaningful benefits [94].

Language barriers and a lack of culturally adapted interfaces further limit global accessibility. The majority of AI tools are developed and deployed in English, posing challenges for non-native speakers and those unfamiliar with technical jargon. Inclusive design features such as multilingual support, culturally sensitive content, and user-friendly visualizations remain rare in current sport technology ecosystems [95].

To narrow the digital divide, several strategies have been proposed. These include the development of open-source AI tools for community sports programs, government subsidies for wearables in schools and grassroots clubs, and public–private partnerships aimed at equitable technology dissemination. Crucially, involving diverse user groups in the design, testing, and implementation of AI systems can help ensure that technologies are responsive to a broader range of needs and contexts [96]. Without targeted design, pricing, and validation policies, AI risks widening existing participation and performance gaps rather than narrowing them.

Taken together, these access considerations define baseline requirements for responsible deployment; the safeguards outlined in the Conclusion (athlete-centered consent and data rights, privacy-by-design and data minimization, transparent/explicable models with uncertainty reporting, routine bias audits, independent field validation, and equitable access pathways) should be treated as non-negotiables. Prioritize low-cost hardware profiles and open-source/community tools with multilingual, accessible UIs and digital-literacy support. Establish public–private programs that subsidize devices and connectivity in under-resourced settings.

## 6. Future Directions and Recommendations

### 6.1. Enhancing Model Performance and External Validity (Generalizability)

We refer to AI-adapted reporting frameworks to guide transparent study design and reporting: TRIPOD + AI (prediction models), CONSORT-AI (randomized trials with AI interventions), and SPIRIT-AI (trial protocols). In this narrative review, these frameworks serve as reference points—rather than formal checklists—to highlight where endurance-sport studies align with or deviate from recommended practice.

Model performance refers to how closely a model’s predictions match observed outcomes in data similar to that used for development—captured by discrimination, calibration, and error metrics (e.g., AUC/F1 for classification; RMSE/MAE for regression; Brier score and calibration slope) [13,14,15].

Despite rapid advances in AI applications for endurance sports, many models still struggle with both performance and generalizability. Contributing factors include biased training datasets, limited longitudinal tracking, and under-representation of diverse athlete profiles. As a result, predictions may work well in narrow cohorts but degrade across ages, sexes, performance levels, devices, or geographic contexts.

Improving model performance begins with better data diversity and quality as well as transparent validation and reporting [13,14,15]. Large, multi-center datasets that include amateur and elite, female and male, younger and older athletes help reduce overfitting and improve external validity. Federated learning decentralized training that preserves data privacy can enable international collaborations without centralizing sensitive data [97].

Static, “snapshot” datasets often miss the dynamic physiology of endurance athletes. Time-series modeling (e.g., RNNs and transformer-based architectures) better captures evolving load, HRV, sleep, and context over time, improving predictions of fatigue, adaptation, or readiness [98].

Cross-population and cross-device transfer also remain challenging. Domain adaptation and transfer learning can bridge the gap by fine-tuning models trained on elite datasets for recreational populations (or harmonizing between devices) using smaller, targeted datasets lowering data-collection burden while preserving predictive performance [99]. Ecological validity increases when models incorporate contextual variables such as weather, altitude, travel, illness, and psychological state. Systems that account for situational factors are more likely to provide actionable recommendations for races, training camps, or high-stress periods.

Active-learning schemes can preferentially sample under-represented strata, while scheduled bias/drift audits, public “model cards/data sheets”, and uncertainty communication (prediction intervals or confidence flags) with human-in-the-loop oversight promote accountable deployment. Collectively, these practices address sampling, validation, and modeling, improving transportability and trustworthiness [14,15,99,100,101,102]. These same practices are directly transferable to endurance sport, where data are siloed across devices, teams, and environments, and they complement the recommendations above [14,15,100,102].

In sum, advancing AI utility in endurance sports requires inclusive, high-fidelity datasets; dynamic time-series approaches; device-aware harmonization; rigorous external/field validation; and close collaboration between data scientists and practitioners. Without these, AI risks being performant for the few rather than impactful for the many.

### 6.2. Multimodal Machine Learning for Integrating Physiological, Biomechanical, Metabolic, and Context Data

Predictive performance in endurance settings improves when models learn multimodally; that is, from heterogeneous, time-aligned streams such as HR/HRV and skin temperature (physiology), IMU/kinematics (biomechanics), CGM with ingestion logs (metabolism–nutrition), psychometrics (RPE, mood), and environment (heat, altitude). In multimodal ML/deep learning, fusion typically follows three patterns: early/feature-level fusion (concatenating synchronized features), intermediate fusion with cross-modal attention or gating between modality-specific encoders, and late/decision-level fusion that aggregates predictions from unimodal models [21,103,104]. Representation-learning objectives (e.g., masked-sequence modeling, contrastive cross-modal alignment) help retain utility when a stream is noisy or partially missing [21,103,104].

Beyond CNN/RNN time-series baselines, Transformer architectures enable token-level alignment across modalities and are more robust to asynchronous windows. Time-series Transformers capture long-range temporal structure, while multimodal Transformers use attention to align and fuse HRV, CGM, IMU, weather, and RPE into shared embeddings [101,103,105]. In endurance applications, such designs have improved the prediction of overload, illness risk, and recovery patterns by learning interactions among sleep, training load, thermal stress, and glycemic dynamics [21,101,103,104].

Field-ready, context-aware personalization requires pipelines that handle real-world imperfections: temporal asynchrony (e.g., continuous HR vs. intermittent nutrition logs), missing/degraded modalities (modality dropout, cross-modal distillation, sequence imputation), and uncertainty (calibrated probabilities/prediction intervals for safer decisions) [21,103,104]. Interpretation should remain anchored to modality-specific validity (e.g., wrist-PPG vs. ECG; IMU-to-GRF inference; CGM physiological lag) using reported agreement metrics such as RMSE/MAE/κ and Bland–Altman limits of agreement [64,65].

To meet latency and privacy constraints in coaching workflows, edge AI (on watch/bike computer/phone) with lightweight models reduces round-trip time and limits cloud exposure of sensitive data [100,106,107]. Interoperability and scale are facilitated by standards-based exchange and device profiles (e.g., HL7 FHIR, IEEE 11073) and open APIs, which simplify cross-platform multimodal fusion and reproducibility [106,107,108]. Privacy-preserving learning (federated learning with secure aggregation, differential privacy) enables collaborative improvement across clubs and teams without centralizing raw athlete data [108].

Overall, multimodal integration is not just an enhancement but a prerequisite for reliable, personalized, and scalable AI in endurance sport provided that findings undergo external/field validation across diverse athlete populations and devices [100,101,103,104,105,106,107,108].

### 6.3. Personalization Beyond the Elite Athlete

Although AI-driven tools in endurance sports have primarily targeted elite athletes, there is increasing awareness of the need to support recreational, older, female, and socioeconomically diverse populations. These groups are significantly underrepresented in the development and validation of AI models, raising concerns about equity and effectiveness in performance support systems [109].

A core limitation lies in the lack of diversity within training datasets. Most AI models are built on physiological and behavioral data from young, male, elite athletes, overlooking the variability in metabolism, recovery, and training adaptations seen in broader populations [110]. Consequently, the resulting recommendations may not translate well to amateur athletes, older adults, or women risking reduced efficacy and perpetuating disparities in access to evidence-based guidance.

In addition, non-elite athletes often face barriers such as limited access to coaching, reduced technological literacy, and sporadic data input. AI tools that assume continuous monitoring or advanced user engagement may therefore be less usable or even misleading in these settings [111]. Usability, affordability, and flexibility must become central design considerations to ensure broader adoption.

Promising approaches are beginning to emerge. Smartphone-based solutions with simplified interfaces, low-cost wearables, and minimal data entry (e.g., HR or step count only) have shown potential to deliver meaningful personalization to the general public [112]. Furthermore, participatory design processes involving end users from underrepresented groups can improve contextual relevance and long-term adherence [113].

In summary, expanding AI personalization beyond elite athletes requires deliberate inclusivity in dataset construction, algorithm training, and system design. By addressing these gaps, AI can evolve from a high-performance niche to a more inclusive tool that enhances training, health, and motivation across the full spectrum of endurance athletes.

### 6.4. Human–AI Collaboration in Coaching

AI is increasingly positioned as a valuable assistant in the coaching process, offering insights into training load, recovery, and performance readiness. However, the most effective applications are those that augment rather than replace human decision-making forming a collaborative, symbiotic relationship between coach, athlete, and machine [22,114].

AI excels at analyzing large volumes of physiological and behavioral data in real time, detecting patterns or anomalies that might escape the human eye. For instance, systems integrating HRV, sleep quality, and subjective wellness scores can alert coaches to early signs of overtraining or illness risk [115]. These outputs, however, gain true value when interpreted within the broader context of the athlete’s psychological state, external stressors, and training goals factors that human coaches are uniquely positioned to assess.

The concept of “centaur coaching” borrowed from human–AI collaboration in chess captures this synergy: the coach contributes contextual judgment and athlete-specific insights, while AI delivers data-driven guidance and trend analysis [116]. This integration not only enhances decision-making accuracy but also fosters trust and engagement, as athletes are more likely to follow recommendations filtered through a trusted human intermediary.

Co-adaptive systems, in which both the coach and the AI system learn from each other, are an emerging direction. For example, adaptive platforms can refine their algorithmic suggestions based on repeated coach overrides, while coaches, in turn, adjust their strategies based on AI feedback. This dynamic loop promotes shared expertise rather than automation, leading to more responsive and individualized support [117].

However, successful human AI collaboration requires digital literacy and a basic understanding of algorithmic logic. Coaches must be aware of the assumptions, limitations, and biases inherent in the AI tools they use. Black-box systems that lack interpretability can hinder trust and result in inappropriate decisions. In this regard, XAI is gaining momentum as a bridge between technological outputs and human understanding [24].

In conclusion, the future of coaching lies not in replacing human judgment, but in designing intelligent systems that amplify it. When well-aligned, human AI collaboration can support more nuanced decision-making, injury prevention, and sustainable athlete development in endurance sports.

### 6.5. Research Gaps and Policy Considerations

Despite the rapid integration of AI into endurance sports, several critical research gaps and policy challenges remain unresolved. Addressing these issues is essential to ensure that AI innovations are scientifically robust, ethically sound, and equitably beneficial across diverse athletic populations.

A primary research limitation is the scarcity of longitudinal, ecologically valid datasets. Most existing AI models in sports science are trained on small, homogeneous cohorts over short durations, limiting their capacity to predict long-term adaptations, injury risk, or performance trajectories in real-world conditions [115,118]. To overcome this, collaborative, multi-site initiatives are needed integrating wearable-derived metrics, training load data, psychological indicators, and health outcomes to build and validate models with broader external validity. A second challenge is the lack of standardization in AI model development and reporting. Variability in sensor precision, data labeling protocols, preprocessing methods, and performance metrics makes it difficult to compare findings across studies [13].

From a policy perspective, data governance and ethical oversight in AI applications remain underdeveloped. Many commercially integrated AI platforms collect sensitive biometric and behavioral data without ensuring users’ informed understanding or control. This raises particular concerns for youth, amateur, or low-literacy populations who may lack the capacity to comprehend how their data are stored, analyzed, shared, or monetized [119]. Comprehensive regulatory frameworks are needed to define responsibilities among developers, coaches, teams, and governing bodies ensuring data protection, transparency, and ethical accountability.

Algorithmic transparency is another pressing concern. As AI increasingly influences training plans, nutritional strategies, and recovery protocols, stakeholders must be able to interpret, contextualize, and challenge system recommendations. Black-box algorithms that provide output without explanation can undermine user trust or lead to inappropriate decisions especially in elite, high-stakes environments [24].

Finally, inclusive and participatory AI design must be prioritized. Many current tools are developed based on data from elite, Western-trained athletes, which risks exacerbating global inequities in access to evidence-based performance tools. Future research and development should involve a broader range of athlete demographics including female, recreational, older, and underrepresented populations to ensure that AI systems are fair, adaptable, and contextually relevant [94].

In summary, the advancement of AI in endurance sports hinges not only on technological innovation, but also on interdisciplinary collaboration, rigorous methodological standards, and robust ethical governance. These pillars will be critical to ensuring that AI fulfills its promise in supporting performance, health, and equity in athletic contexts (Table 3).

### 6.6. Actionable Recommendations for Practice and Research

1. Report and validate transparently.
2. Demonstrate external/field validity before deployment. Test across independent cohorts, devices, environments (heat/altitude), and time windows; include leave-one-device/site-out designs.
3. Quantify and communicate uncertainty. Use calibrated probabilities, prediction intervals, and clear user-facing messaging so coaches/athletes interpret outputs safely.
4. Be device-aware. Validate per modality (e.g., wrist-PPG vs. ECG; IMU-to-GRF) and harmonize cross-device signals; surface limits-of-agreement in dashboards.
5. Use multimodal ML/deep learning robustly. Prefer intermediate fusion (attention/gating) with modality-dropout, cross-modal imputation, and temporal models (Transformers/RNNs) for asynchronous streams.
6. Broaden inclusivity. Recruit diverse athletes (sex, age, level, skin tone, geography) and report stratified performance; avoid training only on elite, homogeneous datasets.
7. Human-in-the-loop by design. Preserve coach override, log overrides to improve models, and provide explainability (e.g., feature attributions) for high-stakes outputs.
8. Minimize data and protect privacy. Apply data-minimization, local/edge processing where possible, and privacy-preserving learning (federated/secure aggregation, differential privacy).
9. Ensure interoperability. Use open schemas/APIs (e.g., HL7 FHIR, IEEE 11073) and shared data dictionaries to enable reproducible, cross-platform workflows.
10. Disclose interests and test independence. Declare vendor ties, enable independent benchmarking, and avoid tying recommendations to commercial incentives.
11. Nutrition-specific caution. Pair CGM with intake/training context; report device error/lag; avoid single-signal prescriptions for fueling.
12. Recovery-specific caution. Combine HRV with sleep, load, and symptoms; avoid single-metric decisions; schedule periodic model recalibration.

## 7. Synthesis: Benefits, Challenges, and Practice Implications

Building on the preceding sections, this synthesis distills the main benefits, persistent challenges, and coach-facing practice implications of AI deployment in endurance settings [13,14,15]. Within endurance sports, applied artificial intelligence converts multidimensional data into actionable insights. Representative applications include recovery prediction from HRV combined with training/sleep/diet logs [6]; course-specific pacing via simulation-based optimization [30,120]; wearable-based biomechanical inference (fatigue classification and estimation of ground-reaction forces/moments) [33,34]; weather-aware performance modeling [42]; and CGM-informed personalized fueling models [27,28].

One of the most notable advantages is individualization. AI systems can deliver tailored recommendations based on each athlete’s evolving physiological and psychological profile. In a 12-week longitudinal cohort of endurance athletes ranging from professional to recreational (*n* = 43; 3572 athlete-days), Rothschild et al. [6] trained ML models on daily heart-rate variability together with training load, sleep, diet and subjective wellness measures to predict next-morning perceived recovery status (AM-PRS) and day-to-day HRV change; group models outperformed an intercept-only baseline (AM-PRS RMSE 11.8 vs. 14.1; HRV-change RMSE 0.22 vs. 0.29), while individualized models showed athlete-specific performance (AM-PRS RMSE range 5.5–23.6; HRV-change 0.05–0.44). A small subset of variables provided most of the predictive power. Similarly, Jianjun et al. [121] developed a hybrid model (gradient boosting + neural networks) that fused physiological inputs (e.g., HRV, oxygen consumption, muscle-activation patterns), psychological constructs (mental toughness, athlete engagement, group cohesion) and contextual training data in a multi-sport cohort (*n* = 480), achieving R^2^ = 0.90 for predicting athlete performance outcomes and outperforming conventional statistical and ML baselines (both R^2^ ≈ 0.77).

Another benefit lies in proactive health and readiness monitoring in endurance cohorts. In recreational runners (*n* = 43) completing an outdoor 5 km time trial, smartwatch-derived features (distance, HR, foot-contact time, cadence, stride length, vertical oscillation) enabled ML regression to estimate instantaneous RPE every 5 s: subject-independent models yielded RMSE 1.8 ± 0.8 RPE points (≈12 ± 6% relative RMSE), while brief individual calibration reduced error to 1.00, 0.66, and 0.45 points using 5%, 10%, and 20% of each runner’s data, respectively [47]. Complementarily, in semi-professional runners (*n* = 8) performing repeated 400 m laps on an outdoor track with wearable IMUs, models trained on trunk/lower-limb signals classified non-fatigued vs. fatigued gait with accuracies between 0.76 and 0.91, demonstrating the feasibility of early fatigue detection from wearable kinematics [63].

Furthermore, continuous feedback provided by commercial, data-driven platforms (e.g., WHOOP, Boston, MA, USA; Supersapiens; Garmin Connect) can support day-to-day decisions; however, the fidelity of consumer sensors is mixed. Methodological guidance for wrist-worn heart-rate validation covering criterion measures (e.g., ECG), protocol design, and error metrics is synthesized by Nelson et al. [12]. Empirically, Cadmus-Bertram et al. [54] evaluated several wrist-worn trackers against ECG during graded treadmill exercise in adults, noting acceptable agreement at rest but widening limits-of-agreement as intensity increased, consistent with motion/PPG limitations. In mode-specific testing, Boudreaux et al. [55] validated multiple wearables during cycling and resistance exercise in healthy adults and reported device- and activity-dependent heart-rate error, with larger deviations during resistance work than steady cycling an important caveat when such signals feed AI models. Collectively, these findings motivate device-aware calibration, cross-device harmonization, and explicit uncertainty handling in AI pipelines.

A central challenge is algorithmic transparency. Many deep learning models operate as “black boxes”, where decision pathways are opaque even to their designers. This lack of interpretability can hinder trust among coaches and athletes, especially when model outputs contradict human expertise [122].

Equally critical are ethical concerns. As AI platforms increasingly collect sensitive biometric data, issues around privacy, consent, and data ownership gain prominence. Floridi et al. [96] highlight five key ethical principles—beneficence, non-maleficence, autonomy, justice, and explicability—that are particularly relevant in sports contexts where data use can directly impact physical health and competitive outcomes. Without strong governance, athletes may lose control over their personal information, especially when third-party commercial vendors are involved [123].

Finally, accessibility and equity remain significant obstacles. While elite athletes often benefit from AI-driven insights and professional support, recreational and lower-income athletes may lack access to such technologies. This “digital divide” can exacerbate performance gaps. As Božić [124] argues, limited availability of AI tools reinforces existing inequalities, while Zallio et al. [95] stresses the need for inclusive, human-centered design.

In endurance sports specifically, recent studies demonstrate concrete gains from applied artificial intelligence: HRV-based recovery prediction in mixed-level endurance athletes [6]; course- and terrain-aware pacing via simulation-based optimization in cycling time trials [30,120]; runner-specific biomechanical inference from wearables, including fatigue classification and estimation of ground-reaction forces and moments [33,34]; weather-aware performance modeling across 1258 endurance races [42]; and CGM-informed personalization for fueling decisions [27,28]. Collectively, these endurance-specific findings support precision, personalization, and proactive management; realizing these benefits at scale will require attention to data quality, model transparency, ethical implementation, and equitable access.

Practically, these gains translate into coach-facing decisions: fueling prescriptions that estimate session-specific carbohydrate targets and hydration adjustments from power/pace, HR drift, and environmental load; heat-mitigation plans that project thermal strain with weather-aware models to set intensity caps, rest ratios, and schedule heat-acclimation microcycles; and pacing strategies that derive course- and weather-informed negative-split targets and critical speed/critical power (CS/CP)-based speed bands via simulation-based optimization. These operationalizations clarify where AI adds tangible value for endurance athletes and coaches.

## 8. Limitations and Interpretive Considerations

This article is a narrative review that provides a structured synthesis rather than a protocol-driven systematic evaluation. Targeted database searches and citation tracking may introduce selection and publication bias; only English-language, peer-reviewed sources were considered. Owing to heterogeneity in populations, sensors, algorithms, outcomes, and metrics, no formal risk-of-bias assessment or meta-analysis was undertaken, and effect sizes across studies are not directly comparable. Many AI studies in endurance settings rely on small or convenience samples, device-dependent signals, and internal (rather than external, field-based) validation, which limits generalizability. Measurement noise and dataset shift can arise from sensor modality (e.g., PPG vs. ECG), CGM lag, IMU placement, firmware updates, and environmental confounders. Accordingly, the synthesis should be interpreted as mapping evidence maturity: items labeled as practice-ready are promising but require context-specific calibration, uncertainty communication, and device-aware external validation; emerging areas warrant larger, preregistered, externally validated studies with standardized outcomes and benchmarks.

## 9. Conclusions

AI is rapidly transforming the landscape of endurance sports, enabling unprecedented personalization in training, recovery, and nutrition. Through ML, real-time analytics, and wearable integration, athletes and coaches can now access dynamic insights that extend far beyond traditional laboratory testing. From glucose monitoring and metabolic profiling to individualized supplementation and predictive recovery algorithms, AI facilitates evidence-based strategies that enhance both performance and long-term health.

Yet, as AI becomes more embedded in sports contexts, important challenges remain. Issues related to model accuracy, algorithmic bias, and generalizability threaten to limit the utility of AI tools across diverse populations. Equally pressing are ethical concerns around data privacy, informed consent, and commercialization, especially for youth, amateur, and recreational athletes who may lack access to oversight or technical literacy.

Priority safeguards include athlete-centered consent and data ownership; privacy-by-design architectures and data minimization; transparent and explainable models with clear communication of predictive uncertainty; systematic bias auditing across sex, age, and performance level; vendor independence with conflict-of-interest disclosure; and equitable access pathways [13,14,15,96,125].

To fully harness AI’s potential in optimizing endurance performance and nutritional support, future efforts must prioritize inclusive data collection, interdisciplinary collaboration, and transparent governance. Crucially, AI should serve as a complement and not a substitute for human expertise, enhancing coaching judgment, supporting athlete autonomy, and promoting sustainable performance outcomes.

As the field moves forward, striking a careful balance between technological innovation, ethical responsibility, and equitable access will be key to ensuring that AI-driven advancements are not only scientifically valid, but also widely beneficial across the spectrum of endurance athletes.

## Figures and Tables

**Table 1:** Applications of Artificial Intelligence in Endurance Sports: Domains, Tools, and Outcomes.

Application Domain	AI Tools/Technologies	Key Benefits	Example Applications	Representative References
Training optimization	ML; predictive modeling	Personalized training plans; load management; injury-risk reduction	Athlete-monitoring platforms; decision-support tools	[6,33]
Metabolic health	Wearables with metabolic analytics; CGM-integrated modeling	Real-time glucose trends; energy-availability estimation; personalized dietary targets	CGM dashboards; metabolic analytics	[10,27,28]
Recovery monitoring	HRV analysis; sleep-tracking algorithms	Fatigue detection; optimized recovery cycles	AI-derived recovery scores; HRV/sleep monitors	[6]
Nutritional personalization	Microbiome-informed ML; CGM-based models; AI diet apps	Individualized fueling; macro-/micronutrient optimization	Dietary apps; microbiome nutrition services	[27,28]
Pacing and race strategy	Simulation-based optimization (optimal control/dynamic programming); real-time decision-support	Optimal pacing; terrain/weather adaptation	Race-pacing tools; virtual coaches	[29,30,31]
Behavioral insights	NLP; sentiment analysis; AI coaching assistants	Mental readiness monitoring; motivation tracking	AI chatbots; mental-coaching tools	[36,37]

AI, artificial intelligence; HRV, heart-rate variability; CGM, continuous glucose monitoring; ML, machine learning; NLP, natural language processing.

**Table 2:** AI-Powered Nutritional Personalization in Endurance Sports: Areas, Tools, Functions, and Potential Benefits.

Nutrition Area	AI Technologies/Tools	Functionality	Potential Benefits	Representative References
Macronutrient Personalization	ML, diet-tracking algorithms	Optimizes carbohydrate/protein/fat intake based on training demands	Improved energy availability, performance, and recovery	[9,27,28]
Micronutrient Profiling	AI-enhanced dietary analysis platforms	Identifies deficiencies based on intake patterns and biomarkers	Early detection of deficiencies, optimized immune and metabolic health	[9]
Gut Microbiome Integration	AI + microbiome sequencing tools	Links gut data with individualized nutrition recommendations	Supports gastrointestinal health, metabolic flexibility	[9,27,28]
Energy Expenditure Estimation	Wearables with metabolic modeling	Estimates caloric needs in real-time based on activity and physiology	Reduces risk of RED-S, enhances fueling strategies	[6]
Hydration and Electrolyte Balance	Smart bottles, sweat sensors + AI	Tracks sweat rate and electrolyte loss, suggests rehydration protocols	Maintains fluid balance, reduces cramping and heat strain	[26]
Behavioral Nutrition Coaching	Conversational AI, digital assistants	Offers motivation, habit tracking, and behavior modification	Improves adherence, educates athletes, supports long-term health	[9,36,37]

AI, artificial intelligence; ML, machine learning.

**Table 3:** Comparison of Conventional vs. AI-Based Approaches in Endurance Sport Support.

Domain	Conventional Approach	AI-Based Approach	Key Advantage of AI	Representative References
Training Load	Coach estimation, RPE logs	Predictive algorithms, real-time data modeling	Data-driven personalization	[6]
Nutrition	Static meal plans	Dynamic, bio-individual diet via app/microbiome AI	Precision and adaptability	[9,27,28]
Recovery	Subjective feedback, HR monitoring	HRV, sleep analysis, strain scores (e.g., WHOOP)	Early fatigue detection, proactive adjustment	[6,12,54,55]

## Data Availability

No new data were created for this manuscript.

## References

[1] Amawi A., AlKasasbeh W., Jaradat M., Almasri A., Alobaidi S., Hammad A.A., Bishtawi T., Fataftah B., Turk N., Saoud H.A. (2024). Athletes’ Nutritional Demands: A Narrative Review of Nutritional Requirements. Front. Nutr..

[2] Cossich V.R.A., Carlgren D., Holash R.J., Katz L. (2023). Technological Breakthroughs in Sport: Current Practice and Future Potential of Artificial Intelligence, Virtual Reality, Augmented Reality, and Modern Data Visualization in Performance Analysis. Appl. Sci..

[3] Zhou D., Keogh J.W.L., Ma Y., Tong R.K.Y., Khan A.R., Jennings N.R. (2025). Artificial Intelligence in Sport: A Narrative Review of Applications, Challenges and Future Trends. J. Sports Sci..

[4] Menges T., Dindorf C., Bartaguiz E., Gassmann F., Fröhlich M. (2024). Advancing Endurance Sports with Artificial Intelligence: Application-Focused Perspectives. Artificial Intelligence in Sports, Movement, and Health.

[5] Lerebourg L., Saboul D., Clémençon M., Coquart J.B. (2023). Prediction of Marathon Performance Using Artificial Intelligence. Int. J. Sports Med..

[6] Rothschild J.A., Stewart T., Kilding A.E., Plews D.J. (2024). Predicting Daily Recovery during Long-Term Endurance Training Using Machine Learning Analysis. Eur. J. Appl. Physiol..

[7] Smaranda A.M., Drăgoiu T.S., Caramoci A., Afetelor A.A., Ionescu A.M., Bădărău I.A. (2024). Artificial Intelligence in Sports Medicine: Reshaping Electrocardiogram Analysis for Athlete Safety—A Narrative Review. Sports.

[8] Nasb M., Zhang Y., Chen N. (2025). The Role of Artificial Intelligence in Precision Exercise Nutrition: A Shift from Data to Diets. Food Sci. Hum. Wellness.

[9] Wu X., Oniani D., Shao Z., Arciero P., Sivarajkumar S., Hilsman J., Mohr A.E., Ibe S., Moharir M., Li L.-J. (2025). A Scoping Review of Artificial Intelligence for Precision Nutrition. Adv. Nutr..

[10] Flockhart M., Larsen F.J. (2024). Continuous Glucose Monitoring in Endurance Athletes: Interpretation and Relevance of Measurements for Improving Performance and Health. Sports Med..

[11] Zeng Z., Liu Y., Hu X., Tang M., Wang L. (2022). Validity and Reliability of Inertial Measurement Units on Lower Extremity Kinematics During Running: A Systematic Review and Meta-Analysis. Sports Med. Open.

[12] Nelson B.W., Low C.A., Jacobson N., Areán P., Torous J., Allen N.B. (2020). Guidelines for Wrist-Worn Consumer Wearable Assessment of Heart Rate in Biobehavioral Research. NPJ Digit. Med..

[13] Collins G.S., Reitsma J.B., Altman D.G., Moons K.G.M. (2015). Transparent Reporting of a Multivariable Prediction Model for Individual Prognosis or Diagnosis (TRIPOD): The TRIPOD Statement. BMJ.

[14] Liu X., Cruz Rivera S., Moher D., Calvert M.J., Denniston A.K., Ashrafian H., Beam A.L., Chan A.-W., Collins G.S., Darzi A. (2020). Reporting Guidelines for Clinical Trial Reports for Interventions Involving Artificial Intelligence: The CONSORT-AI Extension. BMJ.

[15] Cruz Rivera S., Liu X., Chan A.-W., Denniston A.K., Calvert M.J. (2020). Guidelines for Clinical Trial Protocols for Interventions Involving Artificial Intelligence: The SPIRIT-AI Extension. BMJ.

[16] Jordan M.I., Mitchell T.M. (2015). Machine Learning: Trends, Perspectives, and Prospects. Science.

[17] Murphy K.P. (2014). Machine Learning-A Probabilistic Perspective.

[18] Camacho D.M., Collins K.M., Powers R.K., Costello J.C., Collins J.J. (2018). Next-Generation Machine Learning for Biological Networks. Cell.

[19] LeCun Y., Bengio Y., Hinton G. (2015). Deep Learning. Nature.

[20] Russell S., Norvig P. (2021). Artificial Intelligence: A Modern Approach.

[21] Baltrusaitis T., Ahuja C., Morency L.-P. (2019). Multimodal Machine Learning: A Survey and Taxonomy. IEEE Trans. Pattern Anal. Mach. Intell..

[22] Hammes F., Hagg A., Asteroth A., Link D. (2022). Artificial Intelligence in Elite Sports-A Narrative Review of Success Stories and Challenges. Front. Sports Act. Living.

[23] Ali S., Abuhmed T., El-Sappagh S., Muhammad K., Alonso-Moral J.M., Confalonieri R., Guidotti R., Del Ser J., Díaz-Rodríguez N., Herrera F. (2023). Explainable Artificial Intelligence (XAI): What We Know and What Is Left to Attain Trustworthy Artificial Intelligence. Inf. Fusion.

[24] Samek W., Wiegand T., Müller K.-R. (2017). Explainable Artificial Intelligence: Understanding, Visualizing and Interpreting Deep Learning Models. arXiv.

[25] Thuany M., Valero D., Villiger E., Forte P., Weiss K., Nikolaidis P.T., Andrade M.S., Cuk I., Sousa C.V., Knechtle B. (2023). A Machine Learning Approach to Finding the Fastest Race Course for Professional Athletes Competing in Ironman® 70.3 Races between 2004 and 2020. Int. J. Environ. Res. Public Health.

[26] Wang S., Lafaye C., Saubade M., Besson C., Margarit-Taule J.M., Gremeaux V., Liu S.-C. (2022). Predicting Hydration Status Using Machine Learning Models from Physiological and Sweat Biomarkers During Endurance Exercise: A Single Case Study. IEEE J. Biomed. Health Inform..

[27] Zeevi D., Korem T., Zmora N., Israeli D., Rothschild D., Weinberger A., Ben-Yacov O., Lador D., Avnit-Sagi T., Lotan-Pompan M. (2015). Personalized Nutrition by Prediction of Glycemic Responses. Cell.

[28] Mendes-Soares H., Raveh-Sadka T., Azulay S., Edens K., Ben-Shlomo Y., Cohen Y., Ofek T., Bachrach D., Stevens J., Colibaseanu D. (2019). Assessment of a Personalized Approach to Predicting Postprandial Glycemic Responses to Food Among Individuals Without Diabetes. JAMA Netw. Open.

[29] Bach A.F., Alexandersen J., Lundgaard C.B. (2025). A Numerical Design Methodology for Optimal Pacing Strategy in the Individual Time Trial Discipline of Cycling. Sports Eng..

[30] Zignoli A., Biral F. (2020). Prediction of Pacing and Cornering Strategies during Cycling Individual Time Trials with Optimal Control. Sports Eng..

[31] El Dandachi M.-M., Billat V., Palacin F., Vigneron V. (2025). Early Detection of the Marathon Wall to Improve Pacing Strategies in Recreational Marathoners. AI.

[32] Mason R., Pearson L.T., Barry G., Young F., Lennon O., Godfrey A., Stuart S. (2023). Wearables for Running Gait Analysis: A Systematic Review. Sports Med..

[33] Dimmick H.L., van Rassel C.R., MacInnis M.J., Ferber R. (2023). Use of Subject-Specific Models to Detect Fatigue-Related Changes in Running Biomechanics: A Random Forest Approach. Front. Sports Act. Living.

[34] Johnson W.R., Mian A., Robinson M.A., Verheul J., Lloyd D.G., Alderson J. (2021). Multidimensional Ground Reaction Forces and Moments from Wearable Sensor Accelerations via Deep Learning. IEEE Trans. Biomed. Eng..

[35] Cosoli G., Antognoli L., Veroli V., Scalise L. (2022). Accuracy and Precision of Wearable Devices for Real-Time Monitoring of Swimming Athletes. Sensors.

[36] Duan C., Shu Z., Zhang J., Xue F. (2024). Real-Time Prediction for Athletes’ Psychological States Using BERT-XGBoost: Enhancing Human-Computer Interaction. arXiv.

[37] Zhang X., Lin Z., Gu S. (2025). A Machine Learning Model the Prediction of Athlete Engagement Based on Cohesion, Passion and Mental Toughness. Sci. Rep..

[38] Plews D.J., Laursen P.B., Stanley J., Kilding A.E., Buchheit M. (2013). Training Adaptation and Heart Rate Variability in Elite Endurance Athletes: Opening the Door to Effective Monitoring. Sports Med..

[39] Passfield L., Hopker J.G., Jobson S., Friel D., Zabala M. (2017). Knowledge Is Power: Issues of Measuring Training and Performance in Cycling. J. Sports Sci..

[40] Racinais S., Alonso J.M., Coutts A.J., Flouris A.D., Girard O., González-Alonso J., Hausswirth C., Jay O., Lee J.K.W., Mitchell N. (2015). Consensus Recommendations on Training and Competing in the Heat. Br. J. Sports Med..

[41] Nybo L., Rasmussen P., Sawka M.N. (2014). Performance in the Heat-Physiological Factors of Importance for Hyperthermia-Induced Fatigue. Compr. Physiol..

[42] Mantzios K., Ioannou L.G., Panagiotaki Z., Ziaka S., Périard J.D., Racinais S., Nybo L., Flouris A.D. (2022). Effects of Weather Parameters on Endurance Running Performance: Discipline-Specific Analysis of 1258 Races. Med. Sci. Sports Exerc..

[43] Berry S.E., Valdes A.M., Drew D.A., Asnicar F., Mazidi M., Wolf J., Capdevila J., Hadjigeorgiou G., Davies R., Al Khatib H. (2020). Human Postprandial Responses to Food and Potential for Precision Nutrition. Nat. Med..

[44] Bermingham K.M., Linenberg I., Polidori L., Asnicar F., Arrè A., Wolf J., Badri F., Bernard H., Capdevila J., Bulsiewicz W.J. (2024). Effects of a Personalized Nutrition Program on Cardiometabolic Health: A Randomized Controlled Trial. Nat. Med..

[45] Dash S. (2024). Win Your Race Goal: A Generalized Approach to Prediction of Running Performance. Sports Med. Int. Open.

[46] Davidson P., Düking P., Zinner C., Sperlich B., Hotho A. (2020). Smartwatch-Derived Data and Machine Learning Algorithms Estimate Classes of Ratings of Perceived Exertion in Runners: A Pilot Study. Sensors.

[47] Pirscoveanu C.-I., Oliveira A.S. (2024). Prediction of Instantaneous Perceived Effort during Outdoor Running Using Accelerometry and Machine Learning. Eur. J. Appl. Physiol..

[48] Barsumyan A., Shyla R., Saukkonen A., Soost C., Graw J.A., Burchard R. (2025). Quantifying Training Response in Cycling Based on Cardiovascular Drift Using Machine Learning. Front. Artif. Intell..

[49] Wang G., Mao X., Zhang Q., Lu A. Fatigue Detection in Running with Inertial Measurement Unit and Machine Learning. Proceedings of the 2022 10th International Conference on Bioinformatics and Computational Biology (ICBCB).

[50] Chang P., Wang C., Chen Y., Wang G., Lu A. (2023). Identification of Runner Fatigue Stages Based on Inertial Sensors and Deep Learning. Front. Bioeng. Biotechnol..

[51] Radtke T., Crook S., Kaltsakas G., Louvaris Z., Berton D., Urquhart D.S., Kampouras A., Rabinovich R.A., Verges S., Kontopidis D. (2019). ERS Statement on Standardisation of Cardiopulmonary Exercise Testing in Chronic Lung Diseases. Eur. Respir. Rev..

[52] Bowler A.-L.M., Whitfield J., Marshall L., Coffey V.G., Burke L.M., Cox G.R. (2022). The Use of Continuous Glucose Monitors in Sport: Possible Applications and Considerations. Int. J. Sport Nutr. Exerc. Metab..

[53] American Thoracic Society, American College of Chest Physicians (2003). ATS/ACCP Statement on Cardiopulmonary Exercise Testing. Am. J. Respir. Crit. Care Med..

[54] Cadmus-Bertram L., Gangnon R., Wirkus E.J., Thraen-Borowski K.M., Gorzelitz-Liebhauser J. (2017). Accuracy of Heart Rate Monitoring by Some Wrist-Worn Activity Trackers. Ann. Intern. Med..

[55] Boudreaux B.D., Hebert E.P., Hollander D.B., Williams B.M., Cormier C.L., Naquin M.R., Gillan W.W., Gusew E.E., Kraemer R.R. (2018). Validity of Wearable Activity Monitors during Cycling and Resistance Exercise. Med. Sci. Sports Exerc..

[56] Alberti K.G.M.M., Eckel R.H., Grundy S.M., Zimmet P.Z., Cleeman J.I., Donato K.A., Fruchart J.-C., James W.P.T., Loria C.M., Smith S.C. (2009). Harmonizing the Metabolic Syndrome: A Joint Interim Statement of the International Diabetes Federation Task Force on Epidemiology and Prevention; National Heart, Lung, and Blood Institute; American Heart Association; World Heart Federation; International Atherosclerosis Society; and International Association for the Study of Obesity. Circulation.

[57] Zignoli A., Fornasiero A., Stella F., Pellegrini B., Schena F., Biral F., Laursen P.B. (2019). Expert-Level Classification of Ventilatory Thresholds from Cardiopulmonary Exercising Test Data with Recurrent Neural Networks. Eur. J. Sport Sci..

[58] Zignoli A., Fornasiero A., Rota P., Muollo V., Peyré-Tartaruga L.A., Low D.A., Fontana F.Y., Besson D., Pühringer M., Ring-Dimitriou S. (2022). Oxynet: A Collective Intelligence That Detects Ventilatory Thresholds in Cardiopulmonary Exercise Tests. Eur. J. Sport Sci..

[59] Moser O., Riddell M.C., Eckstein M.L., Adolfsson P., Rabasa-Lhoret R., van den Boom L., Gillard P., Nørgaard K., Oliver N.S., Zaharieva D.P. (2020). Glucose Management for Exercise Using Continuous Glucose Monitoring (CGM) and Intermittently Scanned CGM (isCGM) Systems in Type 1 Diabetes: Position Statement of the European Association for the Study of Diabetes (EASD) and of the International Society for Pediatric and Adolescent Diabetes (ISPAD) Endorsed by JDRF and Supported by the American Diabetes Association (ADA). Pediatr. Diabetes.

[60] Sheridan D., Jaspers A., Viet Cuong D., Op De Beéck T., Moyna N.M., de Beukelaar T.T., Roantree M. (2025). Estimating Oxygen Uptake in Simulated Team Sports Using Machine Learning Models and Wearable Sensor Data: A Pilot Study. PLoS ONE.

[61] Hsiao C.-T., Tong C., Coté G.L. (2025). Machine Learning-Based VO2 Estimation Using a Wearable Multiwavelength Photoplethysmography Device. Biosensors.

[62] Meeusen R., Duclos M., Foster C., Fry A., Gleeson M., Nieman D., Raglin J., Rietjens G., Steinacker J., Urhausen A. (2013). Prevention, Diagnosis, and Treatment of the Overtraining Syndrome: Joint Consensus Statement of the European College of Sport Science and the American College of Sports Medicine. Med. Sci. Sports Exerc..

[63] Marotta L., Buurke J.H., van Beijnum B.-J.F., Reenalda J. (2021). Towards Machine Learning-Based Detection of Running-Induced Fatigue in Real-World Scenarios: Evaluation of IMU Sensor Configurations to Reduce Intrusiveness. Sensors.

[64] Miller D.J., Lastella M., Scanlan A.T., Bellenger C., Halson S.L., Roach G.D., Sargent C. (2020). A Validation Study of the WHOOP Strap against Polysomnography to Assess Sleep. J. Sports Sci..

[65] Bent B., Goldstein B.A., Kibbe W.A., Dunn J.P. (2020). Investigating Sources of Inaccuracy in Wearable Optical Heart Rate Sensors. NPJ Digit. Med..

[66] Soligard T., Schwellnus M., Alonso J.-M., Bahr R., Clarsen B., Dijkstra H.P., Gabbett T., Gleeson M., Hägglund M., Hutchinson M.R. (2016). How Much Is Too Much? (Part 1) International Olympic Committee Consensus Statement on Load in Sport and Risk of Injury. Br. J. Sports Med..

[67] Sutehall S., Pitsiladis Y. (2025). Personalized Nutrition for the Enhancement of Elite Athletic Performance. Scand. J. Med. Sci. Sports.

[68] Jäger R., Kerksick C.M., Campbell B.I., Cribb P.J., Wells S.D., Skwiat T.M., Purpura M., Ziegenfuss T.N., Ferrando A.A., Arent S.M. (2017). International Society of Sports Nutrition Position Stand: Protein and Exercise. J. Int. Soc. Sports Nutr..

[69] Peake J.M., Neubauer O., Della Gatta P.A., Nosaka K. (2017). Muscle Damage and Inflammation during Recovery from Exercise. J. Appl. Physiol..

[70] Burke L.M., Sharma A.P., Heikura I.A., Forbes S.F., Holloway M., McKay A.K.A., Bone J.L., Leckey J.J., Welvaert M., Ross M.L. (2020). Crisis of Confidence Averted: Impairment of Exercise Economy and Performance in Elite Race Walkers by Ketogenic Low Carbohydrate, High Fat (LCHF) Diet Is Reproducible. PLoS ONE.

[71] Holzer R., Bloch W., Brinkmann C. (2022). Continuous Glucose Monitoring in Healthy Adults-Possible Applications in Health Care, Wellness, and Sports. Sensors.

[72] Ishihara K., Uchiyama N., Kizaki S., Mori E., Nonaka T., Oneda H. (2020). Application of Continuous Glucose Monitoring for Assessment of Individual Carbohydrate Requirement during Ultramarathon Race. Nutrients.

[73] Liu C., Cao Y., Luo Y., Chen G., Vokkarane V., Ma Y. (2016). DeepFood: Deep Learning-Based Food Image Recognition for Computer-Aided Dietary Assessment. arXiv.

[74] Nogueira-Rio N., Varela Vazquez L., Lopez-Santamarina A., Mondragon-Portocarrero A., Karav S., Miranda J.M. (2024). Mobile Applications and Artificial Intelligence for Nutrition Education: A Narrative Review. Dietetics.

[75] Pfeiffer B., Stellingwerff T., Hodgson A.B., Randell R., Pöttgen K., Res P., Jeukendrup A.E. (2012). Nutritional Intake and Gastrointestinal Problems during Competitive Endurance Events. Med. Sci. Sports Exerc..

[76] de Oliveira E.P., Burini R.C., Jeukendrup A. (2014). Gastrointestinal Complaints During Exercise: Prevalence, Etiology, and Nutritional Recommendations. Sports Med..

[77] Farhud D.D., Zokaei S. (2021). Ethical Issues of Artificial Intelligence in Medicine and Healthcare. Iran. J. Public Health.

[78] Nebeker C., Torous J., Bartlett Ellis R.J. (2019). Building the Case for Actionable Ethics in Digital Health Research Supported by Artificial Intelligence. BMC Med..

[79] Sharon T. (2018). When Digital Health Meets Digital Capitalism, How Many Common Goods Are at Stake?. Big Data Soc..

[80] Canali S., Schiaffonati V., Aliverti A. (2022). Challenges and Recommendations for Wearable Devices in Digital Health: Data Quality, Interoperability, Health Equity, Fairness. PLoS Digit. Health.

[81] Rudin C. (2019). Stop Explaining Black Box Machine Learning Models for High Stakes Decisions and Use Interpretable Models Instead. Nat. Mach. Intell..

[82] Gunning D., Aha D. (2019). DARPA’s Explainable Artificial Intelligence (XAI) Program. AI Mag..

[83] McCradden M.D., Stephenson E.A., Anderson J.A. (2020). Clinical Research Underlies Ethical Integration of Healthcare Artificial Intelligence. Nat. Med..

[84] Tropol E. (2019). Deep Medicine: How Artificial Intelligence Can Make Healthcare Human Again.

[85] Sjoding M.W., Dickson R.P., Iwashyna T.J., Gay S.E., Valley T.S. (2020). Racial Bias in Pulse Oximetry Measurement. N. Engl. J. Med..

[86] Gebru T., Morgenstern J., Vecchione B., Vaughan J.W., Wallach H., Daumé H., Crawford K. (2021). Datasheets for Datasets. arXiv.

[87] Dastin J. (2022). Amazon Scraps Secret AI Recruiting Tool That Showed Bias against Women. Ethics of Data and Analytics.

[88] Seçkin A.Ç., Ateş B., Seçkin M. (2023). Review on Wearable Technology in Sports: Concepts, Challenges and Opportunities. Appl. Sci..

[89] Toner J. (2024). Wearable Technology in Elite Sport: A Critical Examination.

[90] Prainsack B. (2019). Logged out: Ownership, Exclusion and Public Value in the Digital Data and Information Commons. Big Data Soc..

[91] Zuboff S. (2019). The Age of Surveillance Capitalism: The Fight for a Human Future at the New Frontier of Power.

[92] Sujan M., Furniss D., Grundy K., Grundy H., Nelson D., Elliott M., White S., Habli I., Reynolds N. (2019). Human Factors Challenges for the Safe Use of Artificial Intelligence in Patient Care. BMJ Health Care Inform..

[93] van der Sommen F., de Groof J. (2024). Risks and Rewards of AI Democratization. United Eur. Gastroenterol. J..

[94] Obermeyer Z., Powers B., Vogeli C., Mullainathan S. (2019). Dissecting Racial Bias in an Algorithm Used to Manage the Health of Populations. Science.

[95] Zallio M., Ike C.B., Chivăran C. (2025). Designing Artificial Intelligence: Exploring Inclusion, Diversity, Equity, Accessibility, and Safety in Human-Centric Emerging Technologies. AI.

[96] Floridi L., Cowls J., Beltrametti M., Chatila R., Chazerand P., Dignum V., Luetge C., Madelin R., Pagallo U., Rossi F. (2018). AI4People-An Ethical Framework for a Good AI Society: Opportunities, Risks, Principles, and Recommendations. Minds Mach..

[97] Sheller M.J., Edwards B., Reina G.A., Martin J., Pati S., Kotrotsou A., Milchenko M., Xu W., Marcus D., Colen R.R. (2020). Federated Learning in Medicine: Facilitating Multi-Institutional Collaborations without Sharing Patient Data. Sci. Rep..

[98] Vaswani A., Shazeer N., Parmar N., Uszkoreit J., Jones L., Gomez A.N., Kaiser L., Polosukhin I. (2023). Attention Is All You Need. arXiv.

[99] Pan S.J., Yang Q. (2010). A Survey on Transfer Learning. IEEE Trans. Knowl. Data Eng..

[100] Kairouz P., McMahan H.B., Avent B., Bellet A., Bennis M., Bhagoji A.N., Bonawitz K., Charles Z., Cormode G., Cummings R. (2021). Advances and Open Problems in Federated Learning. arXiv.

[101] Zerveas G., Jayaraman S., Patel D., Bhamidipaty A., Eickhoff C. (2020). A Transformer-Based Framework for Multivariate Time Series Representation Learning. arXiv.

[102] Collins G.S., Moons K.G.M., Dhiman P., Riley R.D., Beam A.L., Calster B.V., Ghassemi M., Liu X., Reitsma J.B., van Smeden M. (2024). TRIPOD+AI Statement: Updated Guidance for Reporting Clinical Prediction Models That Use Regression or Machine Learning Methods. BMJ.

[103] Tsai Y.-H.H., Bai S., Liang P.P., Kolter J.Z., Morency L.-P., Salakhutdinov R. (2019). Multimodal Transformer for Unaligned Multimodal Language Sequences. arXiv.

[104] Liang P.P., Zadeh A., Morency L.-P. (2024). Foundations & Trends in Multimodal Machine Learning: Principles, Challenges, and Open Questions. ACM Comput. Surv..

[105] Lim B., Arık S.Ö., Loeff N., Pfister T. (2021). Temporal Fusion Transformers for Interpretable Multi-Horizon Time Series Forecasting. Int. J. Forecast..

[106] Bonawitz K., Eichner H., Grieskamp W., Huba D., Ingerman A., Ivanov V., Kiddon C., Konečný J., Mazzocchi S., McMahan H.B. (2019). Towards Federated Learning at Scale: System Design. arXiv.

[107] McMahan H.B., Moore E., Ramage D., Hampson S., Agüera y Arcas B. (2023). Communication-Efficient Learning of Deep Networks from Decentralized Data. arXiv.

[108] Abadi M., Chu A., Goodfellow I., McMahan H.B., Mironov I., Talwar K., Zhang L. Deep Learning with Differential Privacy. Proceedings of the 2016 ACM SIGSAC Conference on Computer and Communications Security.

[109] Halilaj E., Rajagopal A., Fiterau M., Hicks J.L., Hastie T.J., Delp S.L. (2018). Machine Learning in Human Movement Biomechanics: Best Practices, Common Pitfalls, and New Opportunities. J. Biomech..

[110] Seshadri D.R., Li R.T., Voos J.E., Rowbottom J.R., Alfes C.M., Zorman C.A., Drummond C.K. (2019). Wearable Sensors for Monitoring the Internal and External Workload of the Athlete. NPJ Digit. Med..

[111] Bate G.L., Kirk C., Rehman R.Z.U., Guan Y., Yarnall A.J., Del Din S., Lawson R.A. (2023). The Role of Wearable Sensors to Monitor Physical Activity and Sleep Patterns in Older Adult Inpatients: A Structured Review. Sensors.

[112] Gedi A.A., Khalif A.A., Doon M.A.S., Mohamed A.A., Ali I.A., Ahmed B.A. (2025). Personalized Gym Recommendation System Using Machine Learning. Int. J. Eng. Trends Technol..

[113] Bucher A., Chaudhry B.M., Davis J.W., Lawrence K., Panza E., Baqer M., Feinstein R.T., Fields S.A., Huberty J., Kaplan D.M. (2024). How to Design Equitable Digital Health Tools: A Narrative Review of Design Tactics, Case Studies, and Opportunities. PLoS Digit. Health.

[114] Wu X., Xiao L., Sun Y., Zhang J., Ma T., He L. (2022). A Survey of Human-in-the-Loop for Machine Learning. Future Gener. Comput. Syst..

[115] Claudino J.G., de Oliveira Capanema D., de Souza T.V., Serrão J.C., Machado Pereira A.C., Nassis G.P. (2019). Current Approaches to the Use of Artificial Intelligence for Injury Risk Assessment and Performance Prediction in Team Sports: A Systematic Review. Sports Med. Open.

[116] Haase J. (2025). Augmenting Coaching with GenAI: Insights into Use, Effectiveness, and Future Potential. arXiv.

[117] Lindsay R., Spittle M. (2024). The Adaptable Coach-a Critical Review of the Practical Implications for Traditional and Constraints-Led Approaches in Sport Coaching. Int. J. Sports Sci. Coach..

[118] Gabbett T.J. (2016). The Training-Injury Prevention Paradox: Should Athletes Be Training Smarter and Harder?. Br. J. Sports Med..

[119] Mittelstadt B. (2017). Ethics of the Health-Related Internet of Things: A Narrative Review. Ethics Inf. Technol..

[120] Ashtiani F., Sreedhara V.S.M., Vahidi A., Hutchison R., Mocko G. (2021). Optimal Pacing of a Cyclist in a Time Trial Based on Individualized Models of Fatigue and Recovery. arXiv.

[121] Jianjun Q., Isleem H.F., Almoghayer W.J.K., Khishe M. (2025). Predictive Athlete Performance Modeling with Machine Learning and Biometric Data Integration. Sci. Rep..

[122] Pietraszewski P., Terbalyan A., Roczniok R., Maszczyk A., Ornowski K., Manilewska D., Kuliś S., Zając A., Gołaś A. (2025). The Role of Artificial Intelligence in Sports Analytics: A Systematic Review and Meta-Analysis of Performance Trends. Appl. Sci..

[123] An R. (2025). Artificial Intelligence in Health and Sport Sciences: Promise, Progress, and Prudence. J. Sport Health Sci..

[124] Božić V. (2023). Artifical Intelligence as the Reason and the Solution of Digital Divide. Lang. Educ. Technol..

[125] Guo X., Li X., Guo M. (2024). Diversifying Configurational Paths for Athlete Data Protection. Sci. Rep..

